# Conflicts of Interest in GM *Bt* Crop Efficacy and Durability Studies

**DOI:** 10.1371/journal.pone.0167777

**Published:** 2016-12-15

**Authors:** Thomas Guillemaud, Eric Lombaert, Denis Bourguet

**Affiliations:** 1 Inra, Univ. Nice Sophia Antipolis, CNRS, UMR 1355–7254 Institut Sophia Agrobiotech, Sophia Antipolis, France; 2 Inra, UMR 1062 Centre de Biologie pour la Gestion des Populations (CBGP) Inra-IRD-CIRAD-Montpellier SupAgro, 755 avenue du Campus Agropolis, CS, Montferrier / Lez cedex. France; Universidade Federal de Vicosa, BRAZIL

## Abstract

Public confidence in genetically modified (GM) crop studies is tenuous at best in many countries, including those of the European Union in particular. A lack of information about the effects of ties between academic research and industry might stretch this confidence to the breaking point. We therefore performed an analysis on a large set of research articles (*n* = 672) focusing on the *efficacy* or *durability* of GM *Bt* crops and ties between the researchers carrying out these studies and the GM crop industry. We found that ties between researchers and the GM crop industry were common, with 40% of the articles considered displaying conflicts of interest (COI). In particular, we found that, compared to the absence of COI, the presence of a COI was associated with a 50% higher frequency of outcomes favorable to the interests of the GM crop company. Using our large dataset, we were able to propose possible direct and indirect mechanisms behind this statistical association. They might notably include changes of authorship or funding statements after the results of a study have been obtained and a choice in the topics studied driven by industrial priorities.

## Introduction

Despite the extraordinary financial, political and ideological stakes relating to genetically modified (GM) crops, we were able to identify only two research studies [1, 2] focusing on conflicts of interest (COIs)—defined as “*a set of circumstances that creates a risk that professional judgment or actions regarding a primary interest will be unduly influenced by a secondary interest”* [3]—in this field of research. These two studies reported similar frequencies of COIs in research articles on GM crops, but differed markedly in their conclusions. Sanchez [1] concluded that COIs were not frequent enough to be a serious issue in GM crop studies, whereas Diels *et al*. [2] warned that COIs might be a problem because they were significantly linked to study outcomes. Unfortunately, one of these studies was potentially subject to COI itself [1] (the author’s affiliation was ChileBio, http://www.chilebio.cl, which is funded by companies that develop GM crops) and the other included a limited number of articles [2] (*n* = 94).

**Fig 1:**
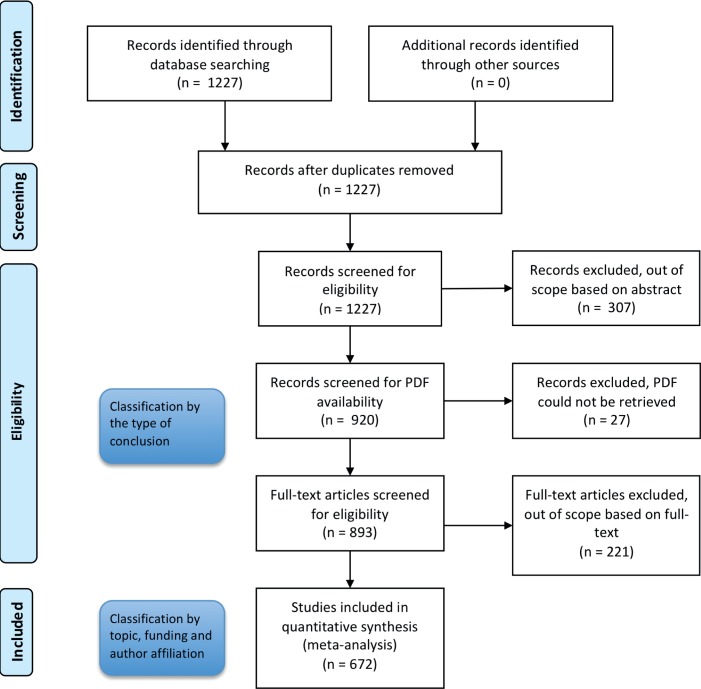
Flowchart diagram describing how literature was gradually included/excluded in the analysis. The details of the protocol of literature inclusion and exclusion are described in the material and methods section.

We investigated the occurrence of COIs in GM crop studies and their association with the outcome of those studies further, by analyzing 672 full-text articles focusing on two topics: the *efficacy* and *durability* of GM crops producing *Bacillus thuringiensis* (*Bt*) toxins–often referred to as *Bt* crops–against target pest insects (see the flowchart of article selection in Fig 1 and S1 Table for a list of all the articles, with the associated classifications).

The *efficacy* and *durability* of *Bt* crops are defined here as the level of pest control provided by the *Bt* toxin they produced or by the crop themselves and the sustainability of this pest control over time, respectively.

We used the COIs created by ties with GM crop companies as a model of COIs for this study because the best interests of these companies was easy to characterize (*efficacy* and *durability* of the crops) and COIs were easy to detect (affiliation of an author to a GM crop company or part of the funding provided by a GM crop company).

## Materials and Methods

### Goal of this study

We explored the occurrence of COIs and the association between COIs and outcomes in studies of genetically modified (GM) crops. We focused our analysis on articles evaluating the *efficacy* or *durability* of GM crops producing toxins (Cry and Vip proteins) from *Bacillus thuringiensis* (*Bt*) against targeted pests. These articles included studies on the main *Bt* crops developed by Monsanto (e.g. maize and cotton varieties expressing the MON810 and MON1076 events, respectively), Syngenta (e.g. maize varieties expressing the Bt11 event), Dow AgroSciences and DuPont Pioneer (e.g. maize varieties expressing the 59122 event).

We used the COI created by the professional affiliation of authors to GM crop companies or the provision of funding for the study by these companies as the model of COI for this study. We considered only COIs linked to GM crop companies because the best interests of these companies were easy to characterize (*efficacy* and *durability* of crops) and the presence of such COIs was easy to detect (an author was affiliated to a GM crop company or some of the funding came from a GM crop company). The COIs investigated here correspond therefore to financial COIs. Non-financial COIs [4], also known as intrinsic COIs or intellectual COIs, were not investigated.

The goal of this study was 1) to measure the extent of COIs in this field of research, 2) to test the hypothesis that study outcomes for the efficacy or durability of Bt crops are more frequently favorable to the interests of GM crop companies in the presence than in the absence of COIs and 3) to investigate the mechanisms underlying the association between COIs and study outcomes.

### Article selection–first step

The flowchart describing the literature included/excluded is shown in Fig 1. We initially selected articles by searching the Web of Science (WoS, ISI Web of Knowledge, Thomson Scientific) bibliographic database. We used a Boolean search term including (1) most crops (e.g. cotton, maize, potato) for which GM varieties producing *Bt* toxins have been commercialized, and (2) most of the pests targeted by *Bt* crops (e.g. *Diabrotica virgifera virgifera*, *Ostrinia nubilalis*, *Pectinophora gossypiella*).

The exact Boolean search term was:

((TI = (rootworm* OR Diabrotica OR borer* OR Ostrinia OR Diatraea OR armyworm* OR Spodoptera OR stalkborer* OR Busseola OR Sesamia OR bollworm* OR Helicoverpa OR earworm* OR Leafworm* OR Pectinophora OR budworm* OR Heliothis OR "Colorado potato beetle" OR "Colorado potato beetles" OR Leptinotarsa OR Chrysomela OR "Cottonwood beetle" OR "Cottonwood beetles" OR "Diamondback moth" OR "Diamondback moths" OR Plutella OR "Rice leaf folder" OR "Rice leaf folders" OR Cnaphalocrocis OR "Chilo suppressalis" OR "Leucinodes orbonalis" OR "Soybean looper" OR "Soybean loopers" OR "Pseudoplusia includens") AND TS = (maize* OR corn* OR "Zea mays" OR cotton* OR "Gossypium hirsutum" OR soybean* OR "Glycine max" OR potato* OR "Solanum tuberosum" OR poplar* OR Populus OR eggplant* OR "Solanum melongena" OR brinjal* OR tomato* OR "Lycopersicon esculentum" OR rice OR "Oryza sativa" OR broccoli* OR "Brassica oleracea" OR tobacco* OR nicotiana) AND TS = ("bt" OR cry* OR bacillus OR toxin*) AND TS = (transgenic* OR GMO* OR GMP* OR "genetically modified" OR "Bt crop" OR "Bt plant" OR "Bt tree")) OR (TS = ("western corn rootworm" OR "Diabrotica virgifera" OR "European corn borer" OR "Asian corn borer" OR "Southwestern corn borer" OR "Ostrinia nubilalis" OR "Ostrinia furnacalis" OR "Diatraea grandiosella" OR "Diatraea saccharalis" OR "Sugarcane Borer" OR "fall armyworm" OR "Spodoptera frugiperda" OR "African maize stalkborer" OR "Busseola fusca" OR "Sesamia nonagrioides" OR "Mediterranean corn borer" OR "Australian bollworm" OR "Helicoverpa punctigera" OR "Helicoverpa zea" OR "Spodoptera littoralis" OR "Corn earworm" OR "Cotton Leafworm" OR "Pink bollworm" OR "Cotton bollworm" OR "Pectinophora gossypiella" OR "Helicoverpa armigera" OR "Tobacco budworm" OR "Heliothis virescens" OR "Colorado potato beetle" OR "Leptinotarsa decemlineata" OR "Chrysomela tremulae" OR "Chrysomela scripta" OR "Cottonwood beetle" OR "Diamondback moth" OR "Plutella xylostella" OR "Rice leaf folder" OR "Cnaphalocrocis medinalis" OR "Striped stem borer" OR "Chilo suppressalis" OR "Leucinodes orbonalis" OR "Fruit Borer" OR "Shoot Borer" OR "Soybean looper" OR "Pseudoplusia includens") AND TI = (maize* OR corn* OR cotton* OR soybean* OR potato* OR poplar* OR eggplant* OR brinjal* OR tomato* OR rice OR broccoli* OR tobacco*) AND TI = ("bt" OR cry* OR bacillus OR toxin*) AND TS = (transgenic* OR GMO* OR GMP* OR "genetically modified" OR "Bt crop" OR "Bt plant" OR "Bt tree")))

We searched the WoS database on September 11, 2015. Using the above search term and limiting the query to articles published in English between 1990 and 2015, we recovered 1,227 articles.

Two of us (DB and TG) then independently screened the titles and abstracts of these 1,227 articles, to eliminate those beyond the scope of our survey. Articles that were classified either with uncertainty by at least one of us (4.6%) or differently (7.9%) were jointly discussed to reach a consensus. We excluded 307 articles which dealt with various subjects, including assessments of the effects of *Bt* crops on non-target organisms, GM crops producing toxins other than those from *Bt* (e.g., toxins from spiders, lectins) and *Bt* crops developed by academics rather than by GM crop companies.

### Classification by the type of conclusion

The outcomes of the 920 articles retained for the analysis were classified as ‘*favorable*’, ‘*neutral*’ or ‘*unfavorable*’ to the financial interests of at least one GM crop company. This classification was done independently by two of us (DB and TG) and was based exclusively on the titles and abstracts of the articles, with no knowledge of the authors’ names and affiliations or of funding sources. This classification was also preformed independently of the classification on the basis of study topic. Articles that were classified either with uncertainty by at least one of us (11.2%) or differently (19.6%) were jointly discussed to reach a consensus.

Articles were classified on the basis of the general tone of the title and abstract and the formal conclusions given in the abstract, on the basis of the following criteria:

1. *Favorable* if the article reported that:
  - One or several of the *Bt* crops tested provided a satisfactory level of control of the targeted pests;
  - The susceptibility of the targeted pest populations to *Bt* toxins was high and/or remained unchanged over time (i.e. indicating an absence of evidence for the selection of mechanisms of resistance to the *Bt* toxins produced by *Bt* crops);
  - Laboratory selection did not yield individuals sufficiently resistant to develop on *Bt* crops;
  - Biotic and abiotic factors (relating to the pests and/or the plants) provided favorable conditions for the management of *Bt* resistance in targeted pests (e.g. *Bt* crops produced high doses of toxins, *Bt* resistance was recessive and/or associated with fitness costs, non-*Bt* crops (called refuges) were planted in sufficient quantities and sufficiently close to *Bt* crops);
  - Pest populations resistant to a particular *Bt* toxin were controlled by another toxin or by other methods for which there was no cross resistance;
  - Several *Bt* toxins acting via different receptors (i.e. decreasing the risk of cross-resistance) could be successfully combined in *Bt* crops through pyramiding.
2. *Neutral* if the article reported:
  - Theoretical models exploring the influence of virtual biotic and abiotic conditions on the selection of *Bt* resistance in targeted pest populations;
  - The development of monitoring tools for following the susceptibility of targeted pest populations to *Bt* crops;
  - The baseline susceptibility of pest populations before the cultivation of *Bt* crops targeting those populations;
  - Receptors for *Bt* toxins and/or mechanisms or genetic changes conferring resistance to these toxins in the targeted pest populations.
3. *Unfavorable* if the articles reported that:
  - One or several *Bt* crops did not provide a high level of control of the targeted pests, potentially jeopardizing the efficacy of the *Bt* resistance management strategy;
  - The susceptibility to *Bt* toxins decreased and/or *Bt* resistance developed in the targeted populations (in either laboratory or natural conditions);
  - Resistance to one *Bt* toxin in the targeted pest populations jeopardized the successful use of other toxins to control those populations (i.e. the occurrence of cross-resistance);
  - Biotic and abiotic factors (relating to the pest and/or the plant) created unfavorable conditions for the management of *Bt* resistance in the targeted pests (e.g. *Bt* crops did not provide high doses of toxins, *Bt* resistance was not fully recessive and/or was not associated with fitness costs, non-*Bt* crops (i.e. refuges) were planted in insufficient quantities and/or too far away from *Bt* crops).

### Article selection–second step

We were able to retrieve PDF files containing the whole content of the article for 893 of the 920 articles classified by the type of conclusion. These 893 PDF files were screened independently by two of us (DB and TG) to check again the relevance of the papers for this study. Articles that were classified either with uncertainty by at least one of us (5.4%) or differently (5.7%) were jointly discussed to reach a consensus. We identified 672 of these articles as relevant (S1 Table).

### Classification by topic

The 672 articles retained were classified into two categories on the basis of the topic considered: the *‘Efficacy’* or *‘Durability’* of the GM crops studied. This classification was done independently by two of us (DB and TG) and was based on titles and abstracts only, blind to the names and affiliations of the authors and to sources of funding (see below). Articles that were classified either with uncertainty by at least one of us (25.1%) or differently (4.3%) were jointly discussed to reach a consensus.

Papers assigned to the *Efficacy* category reported data on:

1. Plant toxicity;
2. Control of the targeted pests;
3. Yield performance;
4. The baseline susceptibility of targeted species;
5. The efficacy of new toxins;
6. New methods of toxin combination;
7. The economic advantages of GM plant use.

Papers assigned to the *Durability* category reported data on:

1. The selection of target pest strains resistant to *Bt* toxins in the laboratory;
2. The initial frequencies of *Bt* resistance alleles within natural populations of the targeted pests;
3. The detection of *Bt* resistance within natural populations of the targeted pests;
4. Changes in the efficacy of *Bt* crops over time;
5. Resistance characteristics (number of genes involved, inheritance, dominance, epistasis, pleiotropy) of the targeted pests;
6. The development of strategies for limiting/preventing the evolution of *Bt* resistance (e.g. the high-dose/refuge strategy) of the targeted pests;
7. Tests of the assumptions underlying such strategies.

### Classification by funding source and author affiliation

We also analyzed the authors’ professional affiliations and the funding sources (see below) of the 672 articles retained for the analysis. This analysis was again performed independently by two of us (DB and TG) blind to the classification of the article for topic and study outcome, as described above. Articles that were classified either with uncertainty by at least one of us (2.1% and 13.7% for authors’ affiliations and the funding sources, respectively) or differently (5.5% and 10.1% for authors’ affiliations and the funding sources, respectively) were jointly discussed to reach a consensus.

For each paper, we recorded:

1. The presence or absence of authors professionally affiliated to a GM crop company. This was established by screening the address of each author appearing in the authors’ affiliation section of the article;
2. For publications co-authored by academics and one or more authors professionally affiliated to a GM crop company, whether the senior author worked for a GM crop company or was an academic researcher. The senior author was identified as the corresponding author for the paper;
3. Whether the sources of funding were declared. In cases in which the funding sources were declared, we distinguished between funding from GM crop companies (partial or complete) and funding from other sources (governmental agencies, non-profit organizations, foundations, universities, etc.).

Using this information, we determined, for each article, whether there was:

1. An *affiliation COI*, defined as the presence of at least one author from a GM crop company on the paper;
2. A *funding COI*, defined as at least partial funding for the study from a GM crop company.

Overall, a paper was considered to display COI if it displayed either an *affiliation* or a *funding COI*.

### Leading authors

For each article, the leading author was identified as the author present on the largest number of papers from among the 672 papers considered in this analysis. If several authors of a given paper had been authors on the same number of articles in our list, the leading author was identified as the first in alphabetic order. We identified 193 leading authors in total (mean number of papers per leading author = 3.48, median = 14.4, minimum = 1, maximum = 53, 5th percentile = 1, 95th percentile = 15)

### Statistical analysis

The associations between (i) the presence/absence of COI and study outcome, (ii) the presence/absence of COI and the topic of the article, and (iii) the topic of the article and its outcome, were assessed in Fisher’s exact tests, with the function fisher.test of R [5].

For a given article, the effect of its topic (i.e. *efficacy* or *durability*) on its number of citations (TC), as recorded on September 11, 2015 in the WoS, was evaluated with a mixed linear model, using the lmer function of R. The data were normalized by log(TC+1) transformation. The number of authors was included as a fixed effect and the identity of the leading author of each article was treated as a random effect. The age of the paper (i.e. 2015 minus the year of publication) was also considered as a fixed effect.

For a given author and a given topic, we evaluated the effect of COI on study outcome, by a generalized linear mixed model approach, with the glmer function of R (library lme4). The topic of the article and COI were considered as fixed effects and the identity of the leading author was treated as a random effect. The outcome of the study was considered as a binary variable with levels ‘*favorable’* and ‘*other’* (containing both ‘*neutral’* and ‘*unfavorable’* categories). We used the Akaike information criterion (AIC) [6] to select the best model.

### Direct and indirect COI links

The global link between COI and the probability of obtaining a positive outcome can be written as follows:

Δ=P(+|COI)−P(+|noCOI)

$$\begin{array}{c} =P\left(t\_1|COI\right)P\left(+|t\_1,COI\right)+P\left(t\_2|COI\right)P\left(+|t\_2,COI\right)\\ -P\left(t\_1|no\;COI\right)P\left(+|t\_1,no\;COI\right)+P\left(t\_2|no\;COI\right)P\left(+|t\_2,no\;COI\right) \end{array}$$

where *t*_1_ and *t*_2_ are the two topics and + within the brackets indicates a positive outcome. In the absence of direct COI links, i.e. when

$$P\left(+|t_{1},COI\right)-P\left(+|t_{1},no\;COI\right)=P\left(+|t_{2},COI\right)-P\left(+|t_{2},no\;COI\right)=0,$$

then

$$\Delta_{\mathrm{indirect}\;\mathrm{link}}=\left(P\left(t_{1}|COI\right)-P\left(t_{1}|no\;COI\right)\right)\left(P\left(+|t_{1},COI\right)-P\left(+|t_{2},COI\right)\right)$$

In the absence of indirect COI links, i.e. when

$$P\left(t_{1}|COI\right)-P\left(t_{1}|no\;COI\right)=0,$$

then

$$\begin{array}{c} \Delta_{\mathrm{direct}\;\mathrm{link}}=P\left(t_{1}|COI\right)\left(P\left(+|t_{1},COI\right)-P\left(+|t_{1},no\;COI\right)\right)\\ +\left(1-P\left(t_{1}|COI\right)\right)\left(P\left(+|t_{2},COI\right)-P\left(+|t_{2},no\;COI\right)\right) \end{array}$$

## Results and Discussion

### A widespread occurrence of COI

**Fig 2:**
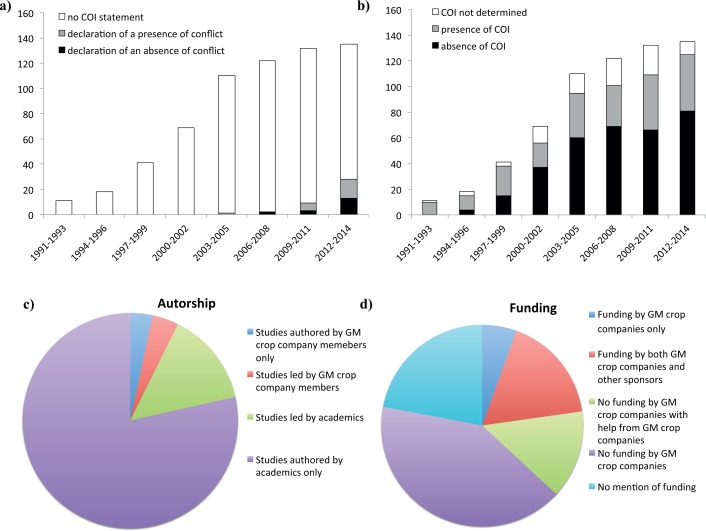
Occurrence of COI in GM crop studies. (**A**) Distribution of declarations of COI over time between 1991 and 2015, in 672 articles focusing on the *efficacy* or *durability* of GM crops producing *Bacillus thuringiensis* toxins against target pest insects; (**B**) Distribution of COI over time between 1991 and 2015; (**C**) Frequencies of the various affiliations; (**D**) Frequencies of funding categories.

Like Sanchez [1] and Diels *et al*. [2] we found that COIs were widespread in the articles considered (Fig 2). Only about 7% (*n* = 46) of the articles contained a declaration of COI, but about one fifth (*n* = 141) of the 672 articles had at least one author from a GM crop company. Details about funding were provided by 524 articles, 29% (*n* = 153) of which acknowledged financial support from a GM crop company. By combining information about authors’ affiliations and funding statements, we were able to assess the presence or absence of COI for a total of 579 articles (Table 1), 40% of which (*n* = 229) presented COIs. This figure is intermediate between those reported by Sanchez [1] (25.8%) and Diels *et al*. [2] (47%).

**Table 1:** Number of articles dealing with the efficacy and the durability of GM *Bt* crops classified according to their topic—durability and efficacy—, their outcome—favorable, neutral or unfavorable to the financial interests of GM crop companies—, and their conflicts of interests (COI)—absence, presence or not determined.

	*Durability*			*Efficacy*		
	Favorable	Neutral	Unfavorable	Favorable	Neutral	Unfavorable
Absence of COI	69	93	83	57	31	17
Presence of COI	42	40	30	81	27	9
COI not determined	6	12	17	33	16	9

### A significant association between COI and study outcome

**Fig 3:**
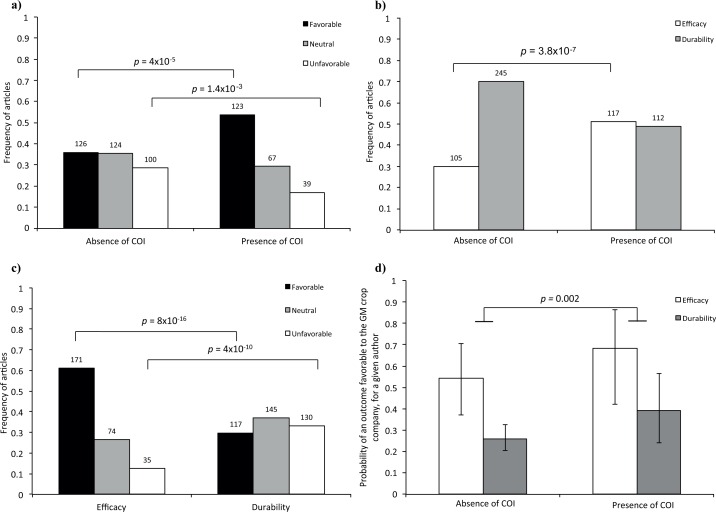
Articles’ outcomes, topics and conflicts of interests. (**A**) Distribution of article outcomes—favorable, neutral or unfavorable to the financial interests of GM crop companies—in the presence (*n* = 229) or absence (*n* = 350) of COI. Note that 93 articles could not be classified for the presence/absence of COI. (**B**) Distribution of the two topics—*durability* and *efficacy*—considered by articles in the presence and absence of COI. (**C**) Distribution of the outcomes of the 672 articles considered, by topic. (**D**) Probability, for a given author, of a paper reporting an outcome favorable to the financial interests of the GM crop company, by topic and the presence or absence of COI. The *P*-values presented are those for Fisher’s exact tests in (a), (b), and (c) and for ANOVA between GLMs in (D). The number of articles is shown above the bars.

For the 579 articles we were able to classify, we found that COIs were associated with a 49% higher frequency of outcomes favorable to the interests of the GM crop company (*p* = 4x10^-5^, Fig 3A). Such an association between COI and study outcome was also reported by Diels *et al*. [2] and has been identified in other fields, including those of tobacco [7], biomedical [8], nuclear [9], sugar [10] and pharmaceutical [11] research. However, the mechanisms underlying this association have been little investigated. We studied these mechanisms in more detail.

The best model describing the outcomes of the studies was that with all fixed (Topic and COI) and random (leading author) effects but no term for interaction between Topic and COI effects. The main factor was Topic, with *efficacy* leading to a favorable outcome in 61% of cases, whereas only 30% of articles on *durability* resulted in a favorable outcome (*p* = 8x10^-16^). The second most important factor was the presence of a COI, which increased the probability of a favorable outcome by +26% for articles on *efficacy* and +50% for articles on *durability*, respectively (*p* = 2.2x10^-3^). The leading author’s identity had an effect of only marginal significance (*p* = 0.07). This model suggests the existence of direct and indirect links between outcome and COI.

### Direct and indirect links between outcome and COI

First, we detected an *indirect* link between outcome and COI through the topics studied. Studies with COIs investigated *efficacy* 70% more frequently than studies without COI (*p* = 3.8x10^-7^, Fig 3B). Moreover, outcomes were 104% more likely to be favorable for articles focusing on *efficacy* than for those exploring *durability* (Fig 3C, *p* = 8x10^-16^). This would naturally lead to, and, therefore, partly explain, the association between COI and favorable outcome.

Second, we found a *direct* link between outcome and COI within each topic. For a given leading author and a given topic, study outcomes were more likely to be favorable to the GM crop company in the presence than in the absence of COI (+26%, *p* = 0.05 for *efficacy*, +50%, *p* = 0.02 for *durability*, and *p* = 2.3x10^-3^ overall, Fig 3D). The statistical effect of COI on outcome (i.e. the difference of probability of obtaining a positive outcome with and without conflict of interest) via this *direct* link (Δ_direct link_ = 0.12) was twice that via the *indirect* link (Δ_indirect link_ = 0.07). Numerical computation of the whole effect, gives Δ = 0.18. Note that Δ_indirect link_ + Δ_direct link_ > Δ simply because of an interaction term.

### A cause or a consequence of COI?

Part of the observed COIs may be due to the favorable outcomes. For instance, authorship or funding statements may be modified after the results of a study have been obtained. GM crop companies may prefer not to be listed as having provided support for studies leading to unfavorable outcomes. Alternatively, academics might be more inclined to propose researchers from GM crop companies as co-authors on publications presenting results favorable to the interests of the company.

**Fig 4:**
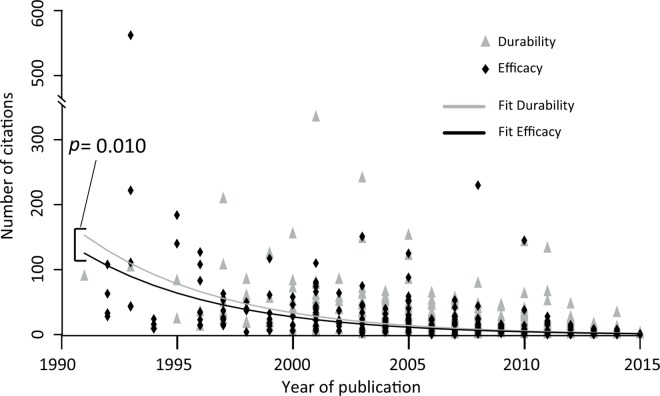
Number of citations (TC) per article (in September 2015), by topic and year of publication. The *p-value* of a common slope for the topics (durability and efficacy) derives from a mixed linear model in which log(TC+1) is fitted to the age of the paper (i.e. 2015 minus the year of publication, *p* = 2x10^-16^), the topic (*p* = 0.010), and the number of authors (*p* = 4.5x10^-6^) as fixed effects and the identity of the leading author of each article as a random effect (*p* = 4.3x10^-9^).

Conversely, COIs may be partly responsible for the favorable outcomes, for at least two reasons: (i) GM crop companies may promote research on *efficacy* rather than *durability*, thereby indirectly increasing the frequency of favorable outcomes for papers with COI. The interests of the scientific community at large seem to be different, as studies without COI focused mostly on *durability*, and on average, peer scientists cited *durability* papers more frequently than *efficacy* papers (between +23% and +51%, *p* = 9.7x10^-3^, depending on the number of authors, the leading author and the year of publication, Fig 4). (ii) Data against the interests of GM crop companies may be less likely to be published in the presence than in the absence of COI. This scientific bias might underlie the *direct* link between favorable outcome and COI found in our study. However, as previously reported by Krimsky [12], this *direct* link is not necessarily the consequence of such a bias. Here, studies displaying COI might have investigated more effective and durable GM crops than those without COI, or they may have used different methodologies (e.g. laboratory vs. field conditions). A closer examination of studies on GM crops is required to determine which of these possibilities actually accounts for the *direct* link between COI and outcome.

### Limitations of this study

This study has several limitations. First, we explored only two characteristics of *Bt* crops: *efficacy* and *durability*. Other characteristics and consequences of these transgenic plants, including all those relating to the environment (e.g. the impact of *Bt* crops on non-target insects) or health, merit a similar analysis. As a demonstration of the absence of such effects, rather than their presence, is clearly in the GM crop companies’ interests, an association between COIs and the outcomes of studies studying environmental or health impacts is also a testable hypothesis. Sanchez [1] and Diels *et al*. [2] focused on studies on health impacts, in which they found COIs to be very frequent, and Diels *et al*. [2] found an association between financial or professional COIs and research outcomes.

Second, as we used the addresses of authors to identify their affiliations, only one type of affiliation, that relating to employment, was considered. However, authors may have affiliations to GM crop companies of other types, such as being members of advisory boards, consultants, or co-holders of patents [13], and this could also have a significant impact on the outcomes of studies on GM crops. We did not consider these affiliations as they are not usually reported in articles (COI statements became obligatory in some journals only recently and, as revealed here, they remain very rare). The consideration of other types of affiliation would require a survey that would be difficult to perform given that more than 1,500 authors were considered in this study.

Third, we have considered only the links between authors and GM crop companies. Other stakeholders (e.g. Greenpeace, The Non-GMO Project, The Organic Consumers Association, The Network of European GMO-free Regions) oppose GM crop companies in being openly against the use of GM crops. An inverse relationship might therefore be expected between the outcomes of studies on GM crops and the presence of COIs relating to these stakeholders. We were unable to test this hypothesis because we identified no financial interests connected with anti-GMO stakeholders, in terms of the professional affiliation of the authors or their declared funding sources.

Finally, this study focused exclusively on financial COIs. Non-financial COIs, also known as intrinsic or intellectual COIs — due to personal, political, academic, ideological, or religious interests — might also have a significant impact on the outcomes of research studies [4]. It is difficult to decipher intellectual COIs and, as for the detection of non-professional affiliations with GM crop companies, it would be a major challenge to perform such an analysis given the large number of authors considered.

We think (although this hypothesis has yet to be tested in this field) that adding studies to overcome these limitations would have resulted in the same qualitative conclusions: COIs are frequent, and there is a link between COIs and study outcomes. It remains unclear whether this would increase the power of the analysis, mostly because some of the abovementioned factors (stakeholders other than GM crop companies, non-professional affiliations to GM crop companies and non-financial COIs) might be difficult to classify and would be subject to considerable background noise.

### Policy implications

Scientific journals should make it obligatory for authors to publish a COI statement, as such statements remain too rare (Fig 2A). However, COI statements are not a panacea and do not absolve scientists and industrial partners of their obligation to deal with COIs [14]. It is almost impossible to avoid non-financial COIs, but it does seem to be possible to limit the occurrence of professional and funding COIs in studies investigating the performance and impact of GM crops. However, it is desirable for these studies to be at least partly funded by GM crop companies, because these companies are the principal beneficiaries of these products and should take responsibility for the impact of their products on the environment and health. One way of combining the possibility of GM crop company funding with an absence of professional and funding COIs would be a system of indirect financial support for research from the industry: GM crop companies and other stakeholders (governments, NGOs) would make a financial contribution to a common pot managed by an independent agency. This agency would launch and fund calls for proposals developed by researchers and stakeholders, to which researchers could respond. Projects would be accepted or rejected by a scientific committee independent of the stakeholders and as free from COIs as possible. With this system, which has already been proposed for biomedical research [15–17], there would be no direct interaction between GM crop companies and researchers: COIs due to professional affiliations and funding sources would be avoided and the continuity of research funding would be independent of the publication of results favorable or unfavorable to the interests of the companies.

## Supporting Information

S1 Table List of the 672 articles with outcomes classified as *favorable*, *neutral* or *unfavorable*, topics categorized as *efficacy* or *durability* and with *affiliation* and *funding COI* characteristics. (XLSX)
